# Social inequalities in traditional and emerging screen devices among Portuguese children: a cross-sectional study

**DOI:** 10.1186/s12889-020-09026-4

**Published:** 2020-06-10

**Authors:** Daniela Rodrigues, Augusta Gama, Aristides M. Machado-Rodrigues, Helena Nogueira, Maria-Raquel G. Silva, Vítor Rosado-Marques, Cristina Padez

**Affiliations:** 1 CIAS – Research Centre for Anthropology and Health, University of Coimbra, Ed. São Bento, Calçada Martim de Freitas, 3000-456 Coimbra, Portugal; 2 Department of Life Sciences, University of Coimbra, Coimbra, Portugal; 3 Department of Animal Biology, Faculty of Sciences of the University of Lisbon, Lisbon, Portugal; 4 High School of Education, Polytechnic Institute of Viseu, Viseu, Portugal; 5 Faculty of Health Sciences, University Fernando Pessoa, Porto, Portugal; 6 Faculty of Human Kinetics, University of Lisbon, Lisbon, Portugal

**Keywords:** Screen time, Television, Mobile devices, Socioeconomic inequalities, Children, Preschool, Portugal

## Abstract

**Background:**

Children are often exposed to too much screen time but few studies have explored the use of old and new digital media among young children. This study assesses screen time, including traditional and mobile devices, in pre-school and elementary school-aged children, according to their gender, age, and socioeconomic position (SEP).

**Methods:**

A total of 8430 children (3 to 10 years; 50.8% boys) from the north, center and south-central Portugal were included in the present study. Data was collected by a parental questionnaire during 2016/2017. Children’s screen time (by media device, weekdays and at the weekend; calculated by mean minutes per day) were reported by parents. Analysis were carried to compare screen time by children’s age, gender and family SEP (classified using father’s educational degree).

**Results:**

Daily screen time was high both in children aged 3 to 5 and 6 to 10 years – 154 min/day (95% CI: 149.51–158.91) and 200.79 min/day (95% CI: 197.08–204.50), respectively – and the majority of children, independently of their gender, exceed the recommended 2 h/day of screen viewing. Children are still primarily engaging in screen time through television but the use of mobile devices, particularly tablets, were already high among 3 year-old children and increased with age. SEP was a negative predictor of screen time in the linear regression analysis, including after adjustment.

**Conclusions:**

Considering the negative health impacts of excessive screen time, recognizing subgroups at risk of excessive screen time and identifying how each device is used according to age is fundamental to enable appropriate future interventions. The screen time in children aged 3–10 years is longer than the recommended, particularly among boys and in those children from lower SEP. Parents and policymakers should have in mind that children spend most of their screen time watching television but mobile devices are becoming extremely popular starting at a young age.

## Background

In developed countries, many children live in a digitally enmeshed world. Time spent with screen media devices, including televisions (TV), computers (PC), electronic video games, smartphones and tablets, saturates the waking hours of young children [1–4]. Specifically, in recent years there has been a rapid uptake of mobile screen media devices among young children living in developed countries [5–7] and a recent study found that over one-half of 3 year olds were given their own tablet [8].

Concerns about the effects of screen time on physical and biopsychological well-being among children have been raised [9–11]. Longer periods exposed to screens have been associated with higher risk of overweight and obesity due to the lack of physical activity and a negative impact on diet [12, 13]. Other potential adverse effects of media exposure includes delayed cognitive and language development, attention deficits and behavioral problems such as violent behavior and aggression [14–16]. Overall, children with higher screen time had increased risk of having a lower well-being, [17] and obesity, depression and anxiety have also been found associated with the use of newer technologies, such as smartphones [18–20]. Studies like those prompted authorities to recommend limits for children’s daily screen: less than 1 h per day for children aged 2 to 5 years and less than 2 h per day for older children [3, 21, 22]. Moreover, the American Academy of Pediatrics [21] encourage parents to develop a family media use plan specific for each family and each family member. However, worldwide, a significant proportion of children are not following the recommended exposure time [23–25].

It is critical to understand when and how young children make use of the different screen media. However, very few studies on screen time have included children below the age of 5 years and most of the findings were based on passive forms of technology prior to the widespread introduction of mobile and touch-screen formats [26, 27]. In addition, identifying population groups with the highest risk of accumulating excessive screen time enables the appropriate targeting of intervention programs. To provide further information on this issue, this study aims to determine the patterns of use of traditional devices (e.g., TV, PC, and electronic video games) as well as emerging devices (e.g., tablet and smartphones) by Portuguese young children according to their gender, age and socioeconomic position (SEP).

## Methods

### Participants

Participants were children aged 3 to 10 years old from three of the largest Portuguese cities (Porto, Coimbra, and Lisbon, respectively located in the north, center and south-central of mainland Portugal) were invited to participate. Data were collected between November 2016 and April 2017 in 118 public and private schools. Participation rates were 60% in Porto, 58% in Coimbra and 67% in Lisbon.

### Variables collected

This study is part of the national project “Inequalities in childhood obesity: the impact of the socioeconomic crisis in Portugal from 2009 to 2015”. The purpose of the referred project is to gain an understanding on the Portuguese prevalence of obesity and to explore a number of possible-related behaviors, including sedentary activities.

The screen-viewing behavior of the children was assessed by parental questionnaires previously used in a similar population [28]. Questionnaires are the most common method to measure screen time, particularly in young children, and the literature suggests that a questionnaire with strong psychometric properties can be a useful tool that estimates screen ownership and time in a simple, fast, no-cost, and completely anonymous way. Specifically, parents were asked to report the average time per day that the child spent watching TV, playing electronic videogames, using the PC, the tablet or a smartphone. Each device was accessed in a different question and separate responses were collected for weekend and weekday use given that previous research suggests that level of children’s screen time may differ between weekdays and weekend [29]. Response options were none = 0, less than 1 h per day (recoded as approximately 30 min), 1 h/d = 60 min, 2 h/d = 120 min, 3 h/d = 180 min, 4 h/d = 240 min, and more than 4 h/d (recoded as approximately 300 min). Total screen time was determined by adding television, computer, video games, smartphone, and tablets use. Given the AAP recommendations for daily screen use [21] and considering that previous studies have found that, for children, ≥2 h daily TV viewing was associated with reduced physical and psychosocial health [30] and <1 h/d of video game playing was associated with positive psychosocial health [31], screen time was categorized into 1) less than 1 h/d, 2) between 1 h/d and 2 h/d, and 3) 2 h/d or more. Items referred to behaviors occurring outside of school hours but did not distinguished recreational from educational use.

Factors such as gender, age, parental education and occupation, and urbanization were collected. Father’s and mother’s educational level was based on the Portuguese Educational system - 9 years’ education or less, 10–12 years’ education (secondary level), and higher education – and grouped into three categories (low, medium, and high). Father’s and mother’s occupation was self-reported and later classified according to the Portuguese Classification of Occupation (CPP/2010), which provided ten groups, namely: 0) Armed Forces, 1) Managers, 2) Professionals, 3) Technicians and associate professionals, 4) Clerical support workers, 5) Service and sales workers, 6) Skilled agricultural, forestry and fishery workers, 7) Craft and related trades workers, 8) Plant and machine operators and assemblers, and 9) Elementary occupations [32]. Place of residence was reported by the parents and urbanization was classified according to the criteria of the Portuguese Statistical System [33]. Father education was used as a proxy measure to the socioeconomic position (SEP), as seen in previous studies, including in the Portuguese context, since the country does not have an official measure of this variable [34, 35].

### Ethical approval

Prior to commencing the study, the protocol was approved by Direção Geral do Ensino (Portuguese Ministry of Education) and Comissão Nacional de Proteção de Dados (CNPD), the Portuguese Data Protection Authority (Authorization number 745/2017). All procedures were in accordance with the 1964 Helsinki declaration and its later amendments. Written informed consent was obtained from children’s parents.

### Statistical analyses

A descriptive analysis was done using the screen time for all 7 days of the week (mean minutes/day in each device) according to gender and SEP, using age as a continuous variable. Later, the sample was divided in two groups according to children’s age: 3 to 5 years (preschool-aged) and 6 to 10 years (elementary school-aged children). Linear regression models with 3 steps were used to predict children’s screen time according to socioeconomic position. Model 2 was adjusted for children’s gender. Model 3, included the variable in previous model, plus mother’s education, parental occupation and urbanization. Model 3 allowed us to explore the interactions of other socioeconomic indicators. The adjusted R—square was determined at each step. A final analysis was done using the categorized screen time and possible statistical differences between age groups were calculated using chi-square tests. All analyses were performed in SPSS version 23 (IBM SPSS® software) using a significance level of 0.05.

## Results

Among the 3 to 5 years old children (*n* = 1860), 52.6% were males and 47.4% were females. The mean age was 4.47 ± 0.67 years. Most preschool-aged children were from medium SEP families (39.5%), followed by high (38.0%) and low SEP (22.5%). A total of 6570 children aged 6 to 10 years old were observed; 50.3% were males and the mean age was 7.94 ± 1.35 years. Most children were from high (37.5%) or medium SEP families (36.9%) (Table 1). Daily total screen time was higher during the weekend than the weekdays, both for younger - 183.15 min/day (95% Confidence Interval: 177.55–188.74) and 97.59 min/day (95% CI: 93.77–101.40), respectively - and older children – 251.61 min/day (95% CI: 247.10–256.12) and 99.91 min/day (95% CI: 97.07–102.75), respectively (data not shown).

**Table 1:** Sample characteristics of Portuguese children participating in the study (2016/2017, *n* = 8430)

	N (%)	3–5 years	6–10 years	*p*-value
Age				
3–5 years	2397 (28.4)	N.A.	N.A.	N.A.
6–10 years	6033 (71.6)	N.A.	N.A.	N.A.
Gender				
Males	4280 (50.8)	1238 (51.6)	3042 (50.4)	0.30
Females	4150 (49.2)	1159 (48.4)	2991 (49.6)	
SEP				
Low	1800 (24.9)	476 (22.6)	1324 (25.9)	0.01
Medium	2705 (37.5)	813 (38.6)	1892 (37.0)	
High	2714 (37.6)	817 (38.8)	1897 (37.1)	

*Legend*. SES: socioeconomic position was calculated by father education level - low (less than 9 years), medium (10 to 12 years), high (university degree); *p*-values calculated by Chi-Square Tests

**Fig. 1:**
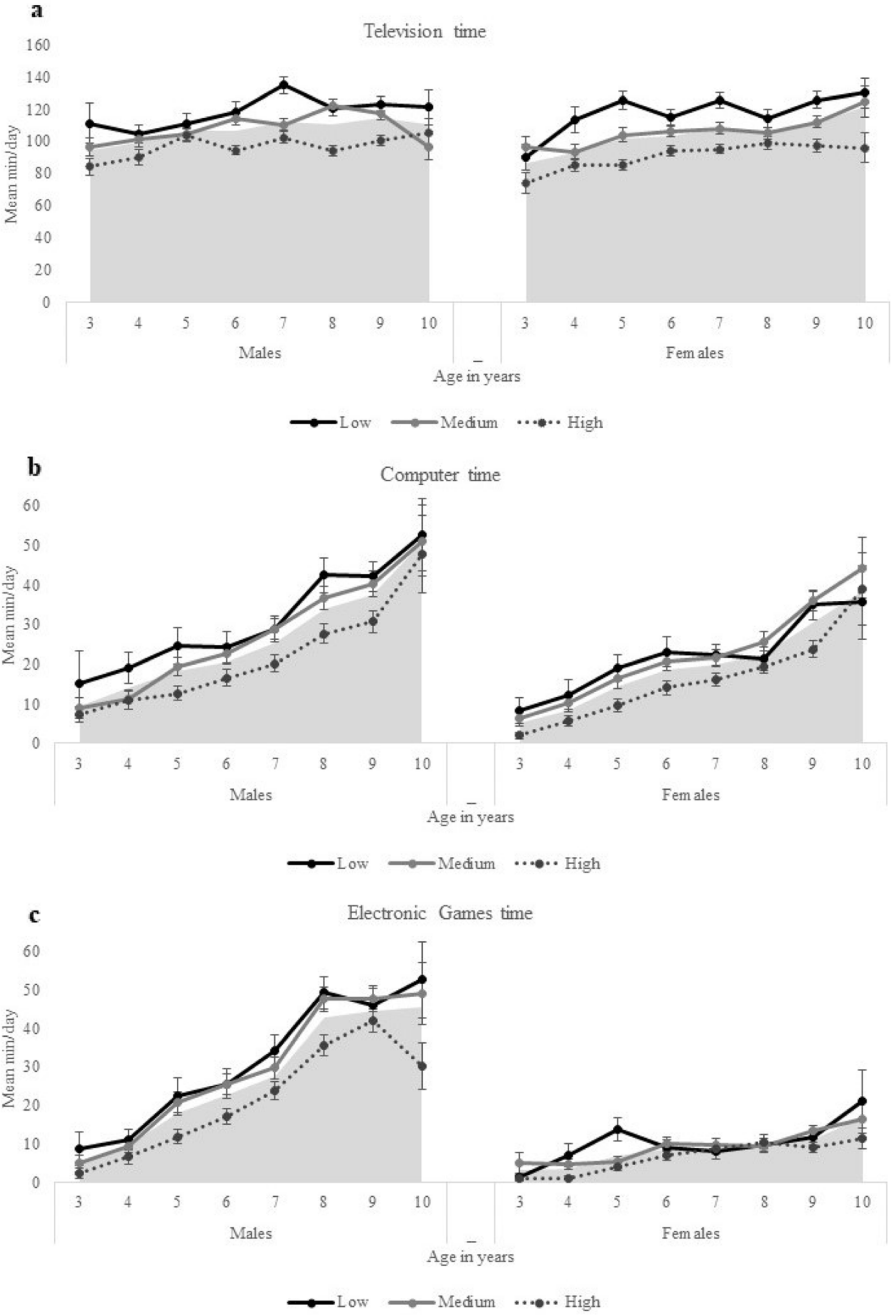
Mean minutes per day spent using (**a**) television, (**b**) computer and (**c**) electronic video games. Legend. Data presented for total screen time (weekdays + weekend) by children’s age, gender, and socioeconomic position defined by the father’s education (low, medium, high). Data for Portugal, 2016/2017. Grey area marks the mean min/day for total sample, according to gender and age. Error bars are custom Standard Error

**Fig. 2:**
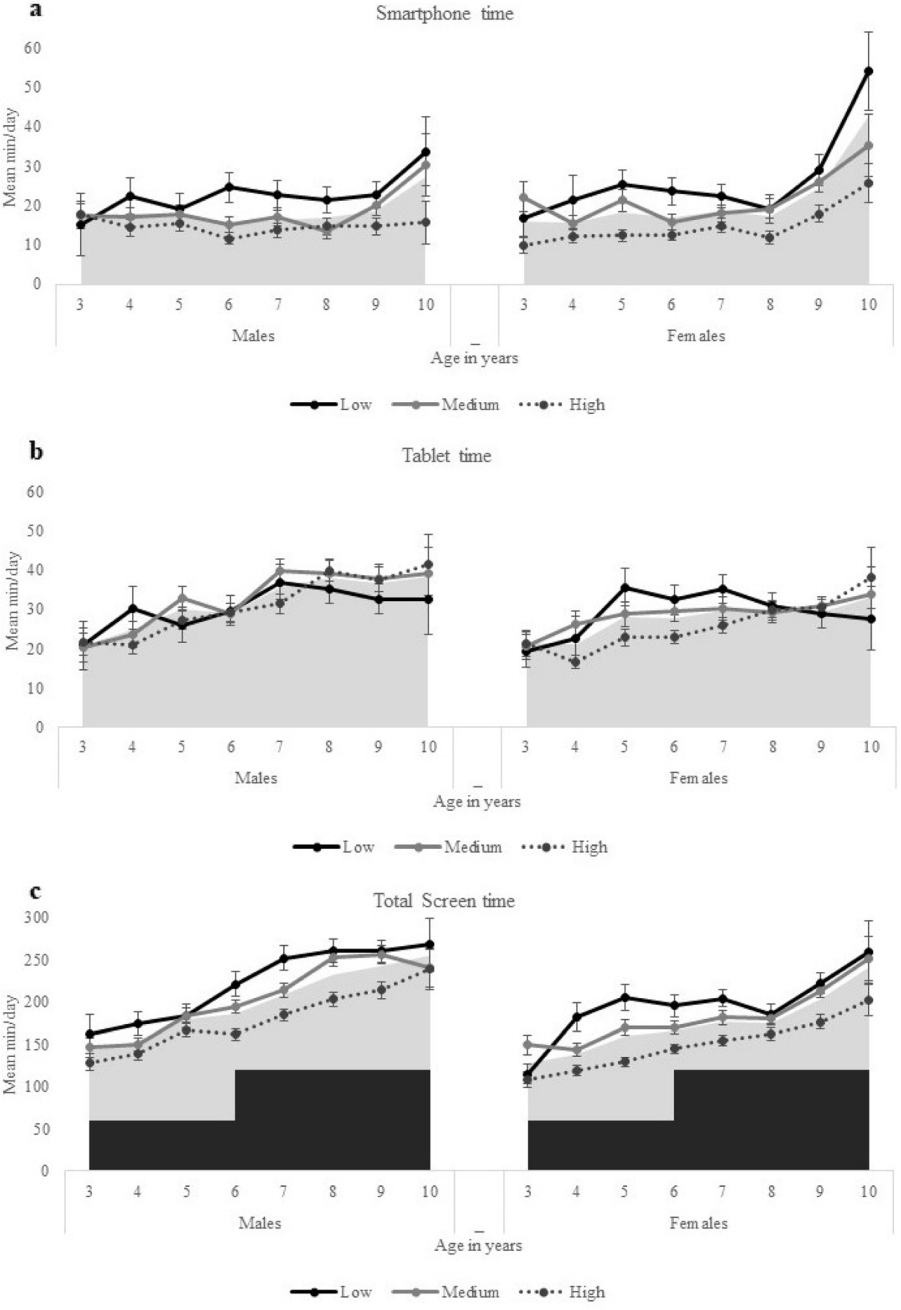
Mean minutes per day spent using (**a**) smartphones, (**b**) tablets and (**c**) all devices combined. Legend. Total screen time includes the use of television, computer, electronic video games, smartphone, and tablet. Data presented for total screen time (weekdays + weekend) by children’s age, gender, and socioeconomic position defined by the father’s education (low, medium, high). Data for Portugal, 2016/2017. Grey area marks the mean min/day for total sample, according to gender and age. Darker area in Fig. 2.c defines the recommended guidelines according to age (<60 min for children 3–5 years and < 120 min for children 6 years and above). Error bars are custom Standard Error

Figures 1 and 2 shows the frequency of weekly use (weekdays plus Saturday plus Sunday) by children’s age and according to their gender and SEP. In comparison with other devices, screen time allocated to TV was the highest, independently of children’s age, remained almost constant across all age groups, and extremely high since a young age. Among girls, the time allocated for computer increased rapidly across ages, while for boys, the most rapidly increase was found for electronic game devices followed by computer. Boys, compared to girls, spent significantly more minutes per day using screen media devices (*p* < 0.001) and differences according to gender are bigger among older children. Overall, socioeconomic disadvantaged children spent significantly more time per day using screen devices than children from medium and high SEP and the screen time of children from medium SEP was more similar with their counterparts from lower SEP than from high SEP. Screen time was progressively higher among older children, primarily driven by more time spent on electronic devices such as computers, electronic video games and tablets.

The linear regression shows that SEP was a negative predictor of screen time, meaning that children from higher SEP were more likely to spend less minutes per day using screen media devices compared with socioeconomic disadvantaged children (Tables 2 and 3). However, for most media devices, SEP lost is significantly association when adjusted for confounding variables such as mother education, parental occupation and urbanization. SEP was particularly associated with children’s screen time on weekends than during weekdays. Independently of the age, 4 to 8% of the variance (in the final model) in children’s screen time was explained by the SEP.

**Table 2:** Linear regression models predicting screen time according to socioeconomic position, children aged 3–5 years

	Model 1			Model 2			Model 3		
	B	95% CI	*R*^2^	B	95% CI	*R*^2^	B	95% CI	*R*^2^
**Television time (mean/day)**									
Weekdays	−5.31**	−8.31; − 2.31	0.006	− 5.31**	− 8.21; − 2.31	0.007	1.40	− 2.74; 5.55	0.019
Weekend	−13.27***	−17.37; − 9.24	0.021	− 13.30***	− 17.37; − 9.24	0.028	− 3.21	−8.80; 2.38	0.042
**Computer time (mean/day)**									
Weekdays	−2.50***	−3.76; − 1.24	0.008	− 2.49***	−3.74; − 1.24	0.021	− 0.56	− 2.30; 1.18	0.025
Weekend	−5.80***	−7.86; − 3.74	0.017	− 5.78***	− 7.82; − 3.74	0.040	−1.77	− 4.59; 1.06	0.048
**Electronic Games time (mean/day)**									
Weekdays	− 2.02***	− 3.00; −1.03	0.009	− 2.02***	− 2.99; − 1.05	0.037	− 0.71	− 2.06; 0.63	0.041
Weekend	−4.13***	−6.00; − 2.26	0.010	−4.10***	− 5.92; − 2.28	0.061	−1.90	− 4.41; 0.62	0.065
**Smartphone time (mean/day)**									
Weekdays	−2.69***	−4.15; −1.23	0.007	− 2.69***	− 4.16; − 1.23	0.006	−0.78	− 2.80; 1.25	0.014
Weekend	−4.15***	−6.30; − 2.01	0.008	−4.16***	−6.31; − 2.01	0.008	− 0.78	− 3.75; 2.18	0.020
**Tablet time (mean/day)**									
Weekdays	−1.86*	− 3.53; −0.19	0.002	− 1.86*	−3.53; − 0.20	0.003	− 1.99	−4.30; 0.32	0.002
Weekend	−3.07*	−5.70; − 0.43	0.002	− 3.11*	− 5.73; − 0.49	0.011	−1.97	− 5.60; 1.66	0.013
**Total screen weekdays (mean/day)**									
	−11.51***	−16.80; − 6.22	0.010	− 11.46***	−16.74; − 6.17	0.012	−4.26	− 11.51; 3.00	0.020
**Total screen weekend (mean/day)**									
	−24.66***	− 32.30; −17.01	0.023	− 24.55***	− 32.16; − 16.93	0.030	−12.71*	− 23.10; − 2.31	0.043
**Total screen 7 days (mean/day)**									
	−19.60***	−26.03; − 13.17	0.021	− 19.50***	− 25.91; − 13.09	0.027	−9.59**	−18.33; − 0.85	0.041

Legend. B: Unstandardized beta coefficient; 95% CI: 95% confidence interval; *R*^2^: adjusted R-square; socioeconomic position calculated by father education; Model 1: crude, Model 2: adjusted for children’s gender, Model 3: like model 2 plus mother education, parental occupation and urbanization; *p < 0.05, **p < 0.01, ***p < 0.001

**Table 3:** Linear regression models predicting screen time according to socioeconomic position, children aged 6–10 years

	Model 1			Model 2			Model 3		
	B	95% CI	*R*^2^	B	95% CI	*R*^2^	B	95% CI	*R*^2^
**Television** time (mean/day)									
Weekdays	−8.76***	−10.61; −6.91	0.020	−8.76***	−10.61; − 6.91	0.020	− 1.57	−4.16; 1.02	0.047
Weekend	−15.21***	− 17.77; − 12.65	0.031	− 15.06***	− 17.63; − 12.50	0.032	− 4.06*	−7.64; − 0.49	0.057
**Computer time (mean/day)**									
Weekdays	−2.34***	−3.35; − 1.33	0.005	− 2.13***	− 3.13; − 1.12	0.019	0.21	−1.20; 1.61	0.031
Weekend	−6.94***	− 8.84; − 5.05	0.012	− 6.37***	−8.24; − 4.50	0.047	− 1.66	−4.27; 0.95	0.059
**Electronic Games time (mean/day)**									
Weekdays	−1.87***	−2.80; −0.94	0.004	−1.73***	−2.65; − 0.82	0.045	0.66	−0.62; 1.95	0.055
Weekend	−4.10***	−5.99; −2.20	0.004	−3.72***	−5.49; −1.95	0.137	−0.24	−2.73; 2.26	0.143
**Smartphone time (mean/day)**									
Weekdays	−2.56***	−3.53; −1.60	0.006	−2.41***	−3.38; −1.45	0.011	0.06	−1.28; 1.41	0.024
Weekend	−5.87***	−7.47; −4.27	0.012	− 5.56***	− 7.16; −3.96	0.019	−0.98	−3.22; 1.26	0.034
**Tablet time (mean/day)**									
Weekdays	−0.71	−1.88; 0.47	0.000	−0.63	−1.81; 0.55	0.001	0.26	−1.40; 1.93	0.005
Weekend	−1.71	−3.70; 0.28	0.000	−1.58	−3.56; 0.41	0.008	−3.12*	−5.94; −0.30	0.009
**Total screen weekdays (mean/day)**									
	−14.91***	−18.80; − 11.02	0.015	−14.95***	− 18.84; − 11.07	0.017	−1.50	−6.92; 3.91	0.042
**Total screen weekend (mean/day)**									
	−33.34***	−39.46; −27.22	0.029	− 33.63***	−39.67; − 27.58	0.053	−13.97**	− 22.44; −5.51	0.073
**Total screen 7 days (mean/day)**									
	−27.81***	−32.81; − 22.82	0.032	− 27.97***	−32.92; − 23.03	0.051	−9.97**	− 16.87; − 3.07	0.076

Legend. B: Unstandardized beta coefficient; 95% CI: 95% confidence interval; *R*^2^: adjusted R-square; socioeconomic position calculated by father education; Model 1: crude, Model 2: adjusted for children’s gender, Model 3: like model 2 plus mother education, parental occupation and urbanization; *p < 0.05, **p < 0.01, ***p < 0.001

**Fig. 3:**
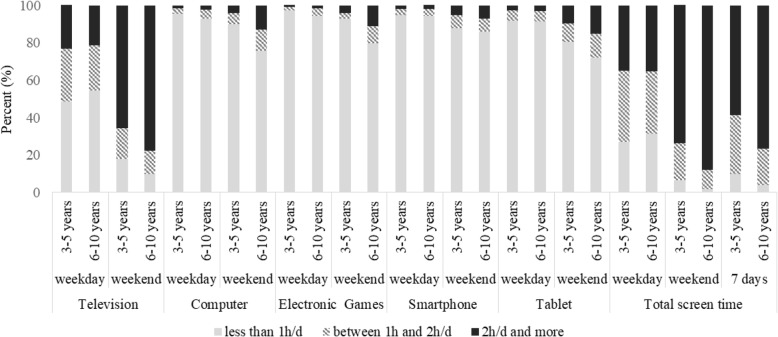
Prevalence of screen time by recommended guidelines for children aged 3–5 years and ≥ 6 years

Figure 3 illustrates the prevalence of screen time by 3 categories. Most children aged 3 to 5 exceed the screen time recommendations (e.g., ≥60 min/day) both during the weekdays (73.1%) and the weekend (93.7%). One in three children aged 6 to 10 years spent 2 h/d or more during school days using screen media devices and 88% exceed that recommendation during the weekend. Television alone accounted for the most screen time with most children spending more than 1 h/d using that device, particularly during the weekend (82.2 and 90.3% for younger and older children, respectively). The prevalence of <1 h daily TV viewing, PC and electronic video games use was significantly lower in older children compared with the ones aged 3 to 5 years (*p* < 0.001) for both weekdays and weekend. The prevalence of ≥1 h/d using tablets and smartphones was significantly higher among older children, compared with their younger counterparts, but differences were only found for the weekend.

## Discussion

This study describes the patterns of use of electronic devices in Portuguese young children, according to their gender, age and SEP, measured by father education. Rapid advancements and increased ownership of information and communications technology in recent years has increased the variety of screen-based media available to young people however, present findings show that TV viewing remains the predominant source of children’s electronic media use. This is consistent with results from other countries like the United States [4, 7, 36] and the United Kingdom [37].

At age 3, children were already using screen base devices on a daily basis and the time spent on screen devices progressively increased with age. This age-related increase in screen time has been commonly reported [37]. However, TV time was already high among 3 and 4 years old children, occupying approximately 91 and 96 min per day, respectively. TV time was similar or even higher than the ones reported in the last decades, before the advent of mobile devices [7, 38, 39] which increases concerns on excessive screen time and potential harmful effects on children’s health [40], given that, the increasingly popularity of new technologies (i.e., tablets and smartphones) are being added to daily traditional screens use (i.e., TV and PC). Chen and Adler (2019) reported a similar pattern in which screen time doubled among children aged 0 to 2 years, from 1997 to 2014, mainly because young children’s TV time did not decrease after the advent of mobile devices [7].

Tablet was the second equipment that consumed more time from children, independently of their age group, gender, and SEP, which is consistent with the latest screen media report carried out in 3–4 and 5–15 years old children from the United Kingdom [41]. Also, a study from North America, found that most children had their own tablet by age 4 and that by age 2, 3 out of 4 children were using mobile devices on a daily basis [8]. Increase ownership and patterns of use suggest that mobile devices, more specifically tablets, may displace TV as major sources of media consumption for young children in the years to come. However, tablets may not be very different from TV, given that a previous study found that photo and video viewing were the most common activities performed on touch-screen devices [42]. In this case, some of the issues that arise with passive watching TV still apply such as, exposure to unsuitable material, passive eating, increased consumption of energy-dense foods and sugar-sweetened beverages, and displacement of other developmentally important activities [43, 44].

Another emerging device, the smartphone, was extremely common, surpassing the use of computers and electronic video game devices among the youngest children. Latest findings show that household ownership of tablets and smartphones doubled in the last decade, including within preschool children [4, 8] which may help to explain present results. Frequent mobile device use is likely to increase children’s social isolation, and hinder opportunities for social interaction with family and friends [45].

In aggregate, the total screen time reported for many of these children, including the young ones, exceed previous recommendations to limit daily screen time to less than 1 or 2 h [21, 22]. In this sample, more than 90% of children spent more than 1 h/day using screen media devices. Moreover, close to 77% of children aged 6 years and above use media devices for 2 h or more on a daily basis which can significantly reduce the physical and psychosocial health [30, 31, 46]. These findings are in line with previous analysis that demonstrated widespread usage of screen-based media in young people living in a variety of European countries, United States, Brazil and Australia, in which at least two thirds of 4 to 17 years old children exceeded 2 h/day of screen time [27, 47]. Similarly, data collected in 2002/2003 among Portuguese children aged 7–9 years showed that approximately 70% spent more than 2 h/day watching TV, which was the most predominant sedentary behavior followed by electronic games and computer use [38].

Consistent with other studies we found that screen time activities differed between boys and girls [23, 47]. Of all the activities covered in this study, gaming had the biggest gender disparity: boys in each age group spend more minutes than girls in a typical week playing games. The same tendency was recently reported for British pre-school and school-aged children [41]. Also, differences by gender increased between age groups and the higher levels of screen viewing by older children could be at least partially explained by increased use of computers and electronic video games [48, 49].

The family SEP was significantly associated with screen time, even after adjusting for confounders, and the disparities were similar to previous studies that found inverse associations between SEP and screen-based media use [7, 47, 50, 51]. SEP explained between 4 to 8% of the variance in children’s screen time which is consistent with the work of Carson and Janssen, [52] where family demographic explained approximately 8% of the variance in screen time among Canadian children aged 0–5 years. Generally, the results indicate that socioeconomic disadvantaged children spent, in average, more time using media devices compared with children from higher SEP. Moreover, in most devices, the results for medium SEP children were more closely related with the ones from low SEP children than with their counterparts from higher SEP. Decreasing costs and marketing strategies can explain the access to screen devices by all SEP groups. In addition, it might be possible that parents from more educated backgrounds set limits when children use screen devices. Other studies have shown that a lower parental education is associated with lower parental modeling, less parental co-viewing, more chance to have a TV in the bedroom and to eat meals in front of the TV [53]. Other possible explanation is that poorer children may spend more time indoors due to a greater likelihood of living in unsafe neighborhoods [54].

The difference in screen media use between lower vs. higher SEP was found between all ages but the gap was even larger in older than in younger children, particularly in some devices like electronic games and smartphones. Present findings suggest the need to intervene at different stages of childhood, starting in young ages, in order to decrease specific screen time behaviors. Physicians and other health care providers should counsel parents and caregivers of young children on the appropriate use of screen time.

Strengths include the large sample size collected at a national level in pre-school aged and schools aged children. In addition, the screen time was obtained for all of the electronic devices, including traditional and emerging/mobile devices such as tablets and smartphones. The survey assessed weekday as well as Saturday and Sunday screen time and the study took both time periods in consideration. Also this was one of the first studies to date to examine screen time in traditional and emerging devices in young children according to the SEP. Limitations of the study include the cross sectional design which makes us unable to draw cause-effect conclusions and to make observations over the time. In addition, since it is not feasible to obtain direct measurements of screen time in large population-based studies, the screen time measures were parental-reported. The information bias associated with these measures may have resulted in an underestimation of screen time.

## Conclusions

This study shows that screen time in children aged 3 to 10 years, is longer than the recommended, particularly among boys, that children engage in these activities starting at a young age and increases in screen-based entertainment use occurs from younger to older children. Moreover, SEP explained some of the variance in children’s screen time, with screen time being higher in socioeconomic disadvantaged children. Effective strategies targeting children and/or their parents are needed to equitably reduce unhealthy behaviors among children.

## Data Availability

The datasets generated during the current study are available from the corresponding author on request.

## Abbreviations

SEP: Socioeconomic position
TV: Television
PC: Computer
EG: Electronic games
d: Day
h: Hour(s)
min: Minutes
SE: Standard error
CI: Confidence interval
AAP: American Academy of Pediatrics
